# Heat Pump Drying of Fruits and Vegetables: Principles and Potentials for Sub-Saharan Africa

**DOI:** 10.1155/2016/9673029

**Published:** 2016-01-06

**Authors:** Folasayo Fayose, Zhongjie Huan

**Affiliations:** ^1^Agricultural and Bio-Resources Engineering, Federal University Oye-Ekiti, PMB 373, Oye-Ekiti 371010, Nigeria; ^2^Tshwane University of Technology, Pretoria, South Africa

## Abstract

Heat pump technology has been used for heating, ventilation, and air-conditioning in domestic and industrial sectors in most developed countries of the world including South Africa. However, heat pump drying (HPD) of fruits and vegetables has been largely unexploited in South Africa and by extension to the sub-Saharan African region. Although studies on heat pump drying started in South Africa several years ago, not much progress has been recorded to date. Many potential users view heat pump drying technology as fragile, slow, and high capital intensive when compared with conventional dryer. This paper tried to divulge the principles and potentials of heat pump drying technology and the conditions for its optimum use. Also, various methods of quantifying performances during heat pump drying as well as the quality of the dried products are highlighted. Necessary factors for maximizing the capacity and efficiency of a heat pump dryer were identified. Finally, the erroneous view that heat pump drying is not feasible economically in sub-Saharan Africa was clarified.

## 1. Introduction

Consumers, in a bid to have healthier and more natural foodstuffs, have been encouraged to increase their daily intake of fruits and vegetables because their nutritional values as suppliers of vitamins, minerals, fiber, and low fat are well recognized. However, the water content of most fruits and vegetables is higher than 80%, which limits their shelf-life and makes them more susceptible to storage and transport problems. Vegetables and fruits can be made more acceptable to consumers by drying [1]. In addition, there is market for dehydrated fruits and vegetables which increases the importance of drying for most of the countries worldwide [2]. In sub-Saharan Africa, a lot of losses of fruit and vegetables are usually experienced during the peak seasons and only a few cold storage of fruits and vegetables is practiced. Although drying is an energy intensive operation, it is highly very indispensable.

According to Bonazzi and Dumoulin [3], drying is needed to extend the shelf-life of foods without the need for refrigerated storage; to reduce weight and bulk volumes, for saving in the cost of transportation and storage; to convert perishable products (surplus) to stable forms (e.g., milk powder); to produce ingredients and additives for industrial transformation (so-called intermediate food products (IFPs), like vegetables for soups, onions for cooked meats, fruits for cakes, binding agents, aroma, food coloring agents, gel-forming and emulsifying proteins, etc.); and to obtain particular convenience foods (potato flakes, instant drinks, breakfast cereals, dried fruits for use as snacks, etc.), with rapid reconstitution characteristics and good sensorial qualities, for special use, such as in vending machines, or directly for consumers. Also, the loss of product moisture content during drying results in an increasing concentration of nutrients in the remaining mass making proteins, fats, and carbohydrates present in larger amounts per unit weight in the dried food than in the fresh.

In the process of drying, heat is required to evaporate moisture from the product and a flow of air to carry away the evaporated moisture, making drying a high energy consuming operation [4]. There are different heat sources available for drying and these have been well discussed in many articles [5]. However, due to the increasing prices of fossils and electricity and the emission of CO_2_ in conventional drying methods, green energy saving and other heat recovery methods for processing and drying of produce become very important. Heat pump technology has been successfully used for drying agricultural products as well as for other domestic dehumidification/heating applications. It has been used for heating, ventilation, and air-conditioning in domestic and industrial sectors in most developed countries of the world including South Africa. However, heat pump drying (HPD) of fruits and vegetables has been largely unexploited in South Africa and by extension to the sub-Saharan African region.

Although studies on heat pump drying started in South Africa several years ago, not much progress has been recorded to date in sub-Saharan Africa. Many potential users view heat pump drying technology as fragile, slow, and high capital intensive when compared with the conventional dryer. However, heat pump drying has been found to be more effective in drying of material with higher amount of free moisture such as tomato [6]. In view of the relevance of heat pump drying, this paper tried to divulge the principles and potentials of heat pump drying technology and the conditions for its optimum use. The paper attempts to bring together the basic information on the effects of heat pump drying, which are inconveniently scattered in several journals and texts in order to justify the need to carry out cutting-edge research on heat pump drying in sub-Saharan Africa.

## 2. Case for Heat Pump Drying Application in Sub-Saharan Africa

Drying to produce high quality agricultural produce especially fruits and vegetables is yet a bottleneck in most sub-Saharan countries, especially Nigeria. Up till now, a lot of food losses are being experienced due to inadequate storage and processing techniques. Heat pump applications are highly required in the sub-Saharan regions of Africa being typical of the warmer regions of the world [7]. Despite the fact that energy failure is a common experience in most sub-Saharan Africa, the use of air conditioning and refrigeration has been on the increase both in the industries as well as in domestic uses because of the prevalent hot conditions of climate in the region. As the use of air conditioning is increasing in this region, so also must the use of heat pump drying be explored. In fact, harnessing of the recoverable heat from these processes which otherwise would have been wasted to some useful purpose would be a worthwhile exercise.

Heat pump drying has also recorded less drying time than other drying methods and it is simple to design [8, 9] making it suitable for low technology countries in the sub-Saharan region. For development of sustainable energy, three important technological changes have been required: energy economies on the demand side, efficiency improvements in the energy production, and renewing of fossil fuels by various sources of renewable energy. In this regard, HPD systems improve energy efficiency and cause less fossil fuel consumption. Since heat pump drying is a low temperature drying process, it will give a double advantage over the conventional, common, and unreliable sun drying in the region. In addition, the fact that the major source of electricity in some sub-Saharan regions including Nigeria is hydropower gives confidence that there is safety to the environment as well as reduced energy costs [10, 11].

Even though heat pump attracts more initial cost, when placed vis-a-vis, the reduced variable electricity cost of running it substantiates its preference to other conventional drying methods. Heat pump dryers are known to be cost effective in many drying applications because it can extract and utilize the latent energy of the air and water vapor for product drying [12]. It has been established that heat pump drying consumes only about half or one-third of the electricity of conventional condenser dryers [7, 13]. Earlier published works in the area of heat pump assisted grain drying found the concept to be mechanically feasible but not attractive economically due to the low fuel prices prevailing at the time [8, 14]. However, Prasertsan and Saen-saby [15] showed that HPD had the lowest operating cost when compared to electrically heated convective dryers and direct-fired dryers.

For heat pump dryers, the total cost of removing a liter of water from a product was observed to be considerably lower at long hours than at short hours of operation. Also Sosle et al. [16] confirmed that HPD is useful for materials with high initial moisture content and in regions with high humidity of ambient air. Therefore, HPD is preferable where high value or quality retention outweighs other considerations. In addition, even though the initial cost of acquiring a heat pump drying set up may be high, yet because of its importance and benefits, the use of heat pump drying technology can be enhanced in developing countries through the assistance of government through incentives, supporting policies and advertising [7]. Moreover, the economic value of purchasing a heat pump depends on the relative costs of the energy types that are consumed and saved.

Jangam and Mujumdar [5] observed that the capital and running costs of heat pumps can be reduced by using heat pumps only over the initial drying period, beyond which the dehumidified drying air does not enhance the drying rate any longer. Also, Mujumdar [17] suggested ways of making heat pump drying technology more cost effective including reducing the capital costs by selecting smaller heat pumps and reducing operational costs by reducing the running time in order to decrease cost of electricity utilization or supplementary use of renewable energy such as solar energy where possible. In addition, heat pumps with multiple modes of heat input and intermittent operation allow the use of smaller heat pumps to service more than a single drying chamber for simultaneous drying of different products.

Also, using mathematical models, concurrent and sequential application of heat by radiation, conduction, and convection can enhance the drying kinetics while improving quality at reduced capital and operating costs. There may be need to switch between different modes of heat input to get the optimum and energy efficient drying condition [18]. Moreover, among the many other observations about heat pump drying that worth future study is the use of the clean water which is gained by condensation. According to* current concerns* [19], the water might be used as a side product. Finally, the heat pump can also be used as cooling plants, which is a basis for further developments towards the cooling and storing of fruit.

## 3. Comparison of Conventional and Heat Pump Drying Processes

Heat pump drying has the ability to recover the latent and sensible heat by condensing moisture from the drying air which may other drying methods cannot do [5]. The recovered heat is recycled back to the dryer through heating of the dehumidified drying air; hence the energy efficiency is increased substantially as a result of heat recovery which otherwise is lost in the atmosphere in conventional dryers [19]. This enables drying at lower temperatures, lower cost, and operation even under humid ambient conditions. A comparison of efficiencies and advantages of heat pump dryers over vacuum and hot air dryers is shown in Table 1. Many articles have been written on the available different types of heat pump dryers [20].

**Figure 1:**
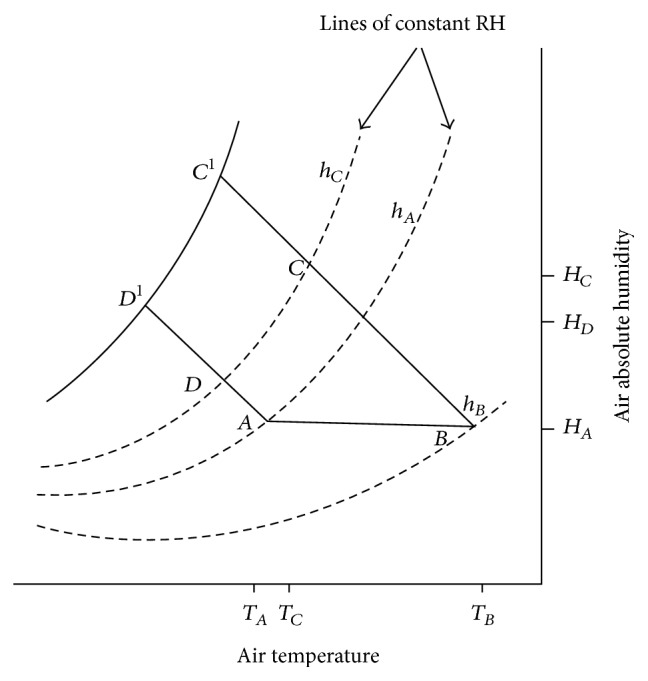
Psychrometric representation of the conventional air drying system. Adapted from FAO [4].

According to FAO [4], the capacity of air to remove moisture depends on its initial temperature and humidity. The relative humidity of the air ought to be controlled so that it does not depend on the absolute humidity of the ambient and the drying temperature [15]. The changes in air conditions when air is heated and passed through a bed of moist product are shown in Figure 1. The heating of air from temperature *T*_*A*_ to *T*_*B*_ is represented by the line *AB*. During heating, the absolute humidity remains constant at *H*_*A*_ whereas the relative humidity falls from *h*_*A*_ to *h*_*B*_. This low relative humidity removes moisture from the materials. As air moves through the material bed it absorbs moisture.

Under adiabatic drying, sensible heat in the air is converted to latent heat and the change in air conditions is represented along a line of constant enthalpy, *BC*. The air will have increase in both absolute humidity, *H*_*C*_, and relative humidity, *h*_*C*_, but fall in temperature, *T*_*C*_. The absorption of moisture by the air would be the difference between the absolute humidities at *C* and *B*, that is, *H*_*C*_ − *H*_*A*_. If unheated air was passed through the bed, the drying process would be represented along the line *AD*. Assuming that the air at *D* was at the same relative humidity, *h*_*C*_, as the heated air at *C*, then the absorbed moisture would be (*H*_*D*_ − *H*_*A*_), considerably less than that absorbed by the heated air (*H*_*C*_ − *H*_*A*_).

**Figure 2:**
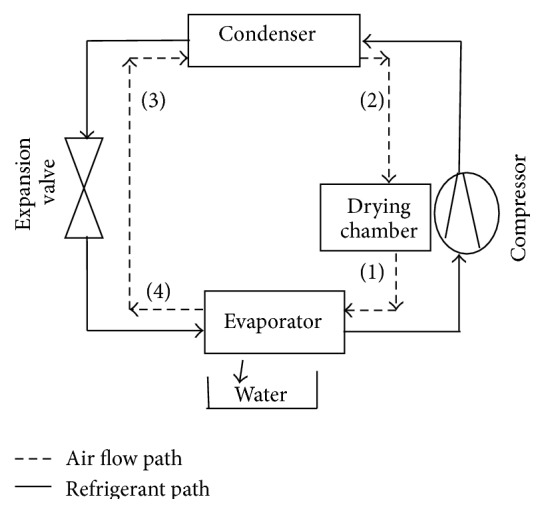
Component arrangement of a heat pump dryer.

At the final stage of drying, there will be little difference of the moisture ratios at the inlet and outlet of the drying chamber. The corresponding temperature difference will also be minimal and these will result in ineffective drying and low thermal efficiency. However, with heat pump drying, there is control of the moisture and temperature of the air as well as heat recovery. In this way, heat pump dryer can improve the product quality while using less energy. The components' arrangement of a typical heat pump drying process is shown in Figure 2. The figure shows that the drying air is dehumidified in the evaporator and reheated to the desired temperature in the condenser before its further passage through the material, thereby offering an advantage of better drying rate and product quality over conventional drying.

## 4. Different Ways of Application of Heat Pump Drying

There are many possible ways of applying heat pump drying. According to Mujumdar and Jangam [20] these possibilities include varying the following: mode of operation, HPD cycle, drying media, supplementary heating, heat pump dryer operation, number of heat pump stages, and temperature for drying. One improvement that heat pump has over other heat sources for drying is that it can be applied to any kind of dryers. Any dryer that uses convection as the primary mode of heat input can be fitted with a suitably designed heat pump, but dryers that require large amounts of drying air, for example, flash or spray dryers, are not suited for HP operation [5, 21]. Heat pump drying technology has been combined with other drying techniques to overcome some problems encountered in those techniques and to achieve improved product quality, reduced energy consumption, high coefficient of performance, and high thermal efficiency [22].

Examples of heat pump assisted drying include heat pump assisted solar drying, microwave drying, infrared drying, fluidized bed drying, atmospheric freeze drying, radiofrequency drying, and chemical heat pump assisted drying [21, 23]. This is in particular with heat sensitive materials like fruits and vegetables that need only low temperature. For example, combining HPD with solar drying enhances the drying and reduces cost. A heat pump is attractive because it can deliver more energy as heat than the electrical energy it consumes. Also it can use modified atmospheres to dry sensitive materials like fruits and vegetables. Moreover, the number of stages of heat pump in the dryer and other arrangements can be varied to improve the performance of the dryer [21]. In addition, chemical heat pump dyer has the advantage of being designed for continuous operation [24] which allows for stable optimum operating conditions [25].

## 5. Quantification of Performances during Heat Pump Drying

According to Yagcioglu et al. [26], the goals of drying in the food industry can be classified into three groups, as follows: (a) economic considerations, (b) environmental concerns, and (c) product quality aspects. Heat pump drying technology achieves all these goals, by drying in environmentally friendly conditions since gases and fumes are not given off into the atmosphere and operates independently outside ambient weather conditions. In addition, energy consumption is drastically reduced (energy cost savings of between 60% and 80%) and quality of products is safely maintained [27]. Also, the condensate can be recovered and disposed off in an appropriate manner, and a potential also exists to recover valuable volatiles from the condensate [13, 23, 28].

The quality attributes of heat pump dried products are presented below.

### 5.1. Quality

The three features of heat pump drying technology that help in controlling quality characteristics include the ability to operate at an absolute humidity less than that of the environment, the ability to select a drying temperature less or higher than the environmental temperature, and the ability to dry in a nonvented chamber by using a modified drying atmosphere [29].

The quality of dried products, as enhanced by heat pump drying, can be comprised by a number of physical, chemical, and sensory characteristics, some of which are discussed below.

#### 5.1.1. Microbial Safety

Quality deterioration caused by microorganisms is undesirable commercially because they limit shelf-life or lead to quality deterioration. Drying helps in reducing or overcoming potential microbial damages. With heat pump drying, microbial safety is minimized by ensuring that all raw materials conform to recognized standards of preparation [29]. Heat pump dryers are able to enhance microbial safety in the foodstuffs by maintaining the relative humidity data at acceptable low level. Also, the operating temperature of heat pump dryers is not limited by the environmental humidity. Moreover, Britnell et al. [30] found that air recirculation in heat pump dryers was not a significant problem for commercial fruit and meat products in Australia, the total bacterial count being typically less than 10^3^ per gram of product. This is because heat pump dryer does not support a large microbial population on the coils or any other site throughout the dryer. However, to guide against microbial activity build-up, sterilization must be done as and when due.

#### 5.1.2. Colour

Colour degradation is a major cause of loss in food drying. The colour of foods is important to their acceptability. Although sulfating agents inhibit enzymatic and nonenzymatic browning (NEB) reactions, their use is surrounded by health and safety concerns. However, enzymatic browning in the drying of peach halves can be reduced by reducing the relative humidity (20%) without the use of sulfites when the moisture content is high (2 kg/kg dry matter) by increasing the air velocity. Mujumdar [17] observed the need to reduce the drying temperature towards the end of the drying cycle in order to avoid NEB. This strategy is relevant to heat pump dryers because the humidity can be controlled independently of the environment.

Also drying of fruits under nitrogen has been found to be effective in inhibiting browning during the initial critical drying period when the moisture content is high. This condition is possible by using modified atmospheric heat pump drying, thereby producing high quality fruits and vegetables. Another way to achieve nonenzymatic browning in banana and other fruits is by using heat pump dryer to produce specific temperature-humidity schedules [29].

#### 5.1.3. Ascorbic Acid Content (AA), Volatile Compound, and Active Ingredients Retention

The impact of constant temperature drying on product quality is well recorded. Most of the product quality parameters, such as NEB and AA content, are often manifested by a progressive loss with increasing temperature. Carrington [29] reported that with proper selection of the temperature schedule, the AA content of the guava pieces can be increased to 20% higher than that in the isothermal drying without significant enhancement in drying time. However, results from Perera and Rahman [28] indicated that using reduced air temperatures at the onset of drying as in the case of heat pump drying, followed by temperature elevation as drying proceeds, yields a better quality product.

Also, the concentration of volatile compound is usually increased by drying, particularly at lower temperatures, typical of heat pump drying. Sunthonvit et al. [31] evaluated the effects of different dryer types, namely, cabinet dryer, tunnel dryer, and heat pump dryer on the composition of volatile compounds of dried nectarine. The result indicated that heat pump dryer is the best system for the preservation of volatile compounds in sliced dried fruits in terms of lactones and terpenoids amongst the three methods of drying. Also, the retention of total chlorophyll content and ascorbic acid content in sweet green pepper was observed by Pal et al. [32] to be more in heat pump-dried samples with higher rehydration ratios and sensory scores than in those hot air-dried.

#### 5.1.4. Aroma and Flavour Loss

Drying methods that employ low temperature do provide high concentration of key aroma compounds [12]. Ginger dried in a heat pump dryer was found to retain over 26% of gingerol, the principal volatile flavour component responsible for its pungency when compared with the rotary dried commercial samples that have only about 20% [29]. The higher volatile retention in heat pump-dried ginger may be due to reduced degradation of gingerol when low drying temperatures are used instead of high convention dryer temperatures. When HPD is conducted in a closed chamber, any compound that volatilizes will remain within it, and the partial pressure for that compound will gradually build up within the chamber, retarding further volatilization from the product [28]. In addition, Carrington [29] observed that the color and aroma herbs (e.g., parsley, rosemary, and sweet fennel) can be improved with HPD when compared with other commercial products. Also, the sensory values of heat pump dried herbs will be nearly doubled when compared with commercially dried products.

#### 5.1.5. Viability

Drying with oxygen-sensitive materials, such as flavor compounds and fatty acids, can undergo oxidation, giving rise to poor flavour, colour, and rehydration properties. Cardona et al. [33] studied the heat pump dehydration of lactic acid bacteria (LAB) to determine the optimum procedure and drying conditions at which LAB can be dehydrated in a heat pump dryer that will not result in unacceptable deterioration of viability and activity. The result of the study indicated that heat pump dehydration of LAB gave favourable results which is comparable with the situation when freeze drying method, which is more costly when used. In addition, use of modified atmospheres obtainable with heat pump drying to replace air would allow new dry products to be developed without oxidative reactions occurring [28], thereby producing seed with high proportion of products with germination potentials.

#### 5.1.6. Rehydration

During drying, important changes in the structural properties of fruits and vegetables can be observed as water is removed from the moist material. Rehydration is a process of moistening dry food materials. In most cases, dried foods are soaked in water before cooking or consumption, therefore rehydration is a very important criterion. Factors affecting the rehydration process include porosity, capillarity and cavity near the food surface, temperature, trapped air bubbles, amorphous-crystalline state, soluble solids, anion, pH of the soaking water, and dryness level. Faster rehydration had been attributed to apple slices dried with a modified atmosphere heat pump dryer [34]. Also, in another study, heat pump and microwave vacuum-dried tomato slices showed comparatively better rehydration ratios than the hot air- or solar cabinet-dried slices [35].

#### 5.1.7. Shrinkage

Shrinkage occurs first at the surface and gradually moves to the bottom of drying objects with an increase in the drying time. Also, the cell wall becomes elongated and cracks are formed in the inner structure as drying proceeds at high temperature. From microscopy, it was found that shrinkage of apple slices dried in convection is significantly anisotropic, while less damage to the cell structure during freeze drying leads to more isotropic deformation [36]. Heating produces major changes in structure of products. Shrinkage occurs because polymer food stuffs cannot support their weight and, therefore, collapse under gravitational force in the absence of moisture. Heat pump drying however involves drying at low temperature, making shrinkage less pronounced.

### 5.2. Drying Efficiency

The performance of a dryer or drying system is characterized by various indices, including energy efficiency, thermal efficiency, volumetric evaporation rate, specific heat consumption, surface heat losses, and unit steam consumption which were defined to reflect the particularities of various drying technologies [37]. Energy efficiency, the ratio of the energy required (Er) to the energy supplied (Es) in drying, is very important because energy consumption is a very significant factor of drying costs [5]. Due to the complex relationships of the food, the water, and the drying medium, that is, the air, a number of efficiency measures can be worked out, each appropriate to circumstances and therefore selectable to bring out special features important in the particular process.

Efficiency calculations are useful when assessing the performance of a dryer, looking for improvements, and in making comparisons between the various classes of dryers which may be alternatives for a particular drying operation [38]. Energy efficiencies are meant for providing an objective comparison between different dryers and drying processes.

There are three groups of factors affecting drying efficiency [4]. They include

- those related to the environment, in particular, ambient air conditions;
- those specific to the crop;
- those specific to the design and operation of the dryer.

The factors relating to the environment are well taken care of by HPD, making it have higher efficiencies than other drying methods [39].

Air-drying efficiency, *η*, can be defined by

(1) $$\begin{array}{c} \eta =\frac{\left(T_{1}-T_{2}\right)}{\left(T_{1}-T_{a}\right)}, \end{array}$$

where *T*_1_ is the inlet air temperature into the dryer, *T*_2_ is the outlet air temperature from the dryer, and *T*_*a*_ is the ambient air temperature. As mentioned earlier, the numerator is a major factor that determines drying efficiency. Also, in order to maximize the efficiency, Strommen and Eikevik [25] suggested that the inlet temperature of the dryer should be maximized in accordance with the product requirement. In addition, energy efficiency is the ratio of the latent heat of evaporation of the moisture removed to the drying air heat input. For HPD systems, drying efficiency is a measure of the quantity of energy used to remove one unit mass of water from the product, normally expressed in kJ kg/water or kWh kg/water.

### 5.3. Specific Moisture Extraction Ratio (SMER)

An alternative indicator of the energy efficiency for heat pump dryers is the specific moisture extraction ratio which is determined using

(2) $$\begin{array}{c} \text{SMER}\left(\;\text{kg/kWh}\;\right)≔\frac{\text{Amount of water evaporated}}{\text{Energy used}}. \end{array}$$

The SMER can be calculated either as an instantaneous value or as an average value during drying [40]. During the drying process, the SMER value decreases as the removal of moisture becomes more difficult, due to smaller water vapor deficits on the surface of the product. For heat pump dryers, SMER value can be above the theoretical maximum value. The energy efficiency of HPD can be reflected in the high SMER values and drying efficiency when compared to other drying systems, as shown in Table 1. Consequently, high SMER would then be translated to low operating cost, making the payback period for initial capital considerably short. Other definitions of specific moisture extraction rate with respect to the compressor power are reported by [29]. Also, according to Strommen and Eikevik [25], the refrigeration capacity should not be oversized so as not to reduce the relative humidity and a consequent reduced SMER.

### 5.4. Coefficient of Performance (COP)

The efficiency of the HPD is indicated by compressor cooling coefficient of performance [12]. COP can be used to evaluate the amount of work converted into heat for two different system operations: cooling and heating. For a heat pump, the heat transfer ${\dot{Q}}_{\text{out}}$ from the system to the hot body is desired, and the coefficient of performance is expressed as

(3) COPhpDesired OutputRequired Input=Heat addedWork required=Q˙outW˙cycle⁡,

where ${\dot{W}}_{cycle}$ is the electrical power input of the compressor.

As much as possible, the evaporating and condensing temperatures should be selected in order to optimize the product of COP and the thermal efficiency [25].

### 5.5. Drying Rate

In air drying, the rate of removal of water depends on the conditions of the air, the properties of the food, and the design of the dryer. Drying rate is expressed as follows:

(4) $$\begin{array}{c} \text{DR}=\frac{m_{t}-m_{t+\Delta t}}{\Delta t}, \end{array}$$

where *m*_*t*_ is the mass at time *t*. Drying rates would decrease as moisture content decreases [38].

Factors affecting the drying rate will vary slightly depending upon the type of drying system used. To attain maximum drying capacity, Strommen and Eikevik [25] enunciated that the air flow should be countercurrent instead of cross flow or cocurrent to the product movement in order to maximize the relative humidity at the dryer's outlet. In addition, Wilhelm et al. [41] suggested the following factors to be considered:

(1) nature of the material: physical and chemical composition, moisture content, and so forth;
(2) size, shape, and arrangement of the pieces to be dried;
(3) wet-bulb depression or relative humidity or partial pressure of water vapor in the air (all are related and indicate the amount of moisture already in the air);
(4) air temperature;
(5) air velocity (drying rate is approximately proportional to *u* ^0.8^).

In general, the drying rate decreases with moisture content, increases with increase in air temperature or decreases with increase in air humidity. At very low air flows, increasing the velocity causes faster drying, but at greater velocities the effect is minimal indicating that moisture diffusion within the grain is the controlling mechanism [38].

### 5.6. Specific Energy Consumption

Specific energy consumption depends on the dryer efficiency, the product, and the initial moisture content. The moisture near the surface of products needs more energy for its removal than the moisture at the center of the product. This is because the moisture flows from the center to the surface. The specific energy consumption is estimated by considering the drying time involved and energy utilization by the various components of the dryer. It is expressed in terms of MJ/kg of water removed and used as one of the factors in the optimization of process parameters. According to Jokiniemi et al. [14], it can be calculated by integrating the energy used and by calculating the amount of moisture removed as in

(5) $$\begin{array}{c} Qs=\frac{Qh}{\Delta G}, \end{array}$$

where *Qs* is the specific energy consumption of drying; *Qh* is the energy consumption of drying air; and Δ*G* is the mass of the evaporated water.

## 6. Conclusion

This paper has reviewed the principles and potentials of heat pump drying of fruits and vegetables for application to sub-Saharan Africa. It has been shown in this paper that heat pump dryers are promising technologies that maintain product quality and reduce energy consumption of drying, particularly for high value products like fruits and vegetables. The application of heat pump drying contributes positively to the following fruit and vegetables quality attributes including improved microbial safety, better colour, vitamin C retention, enhanced volatile compound, aroma and flavor compounds, rehydration, and texture. Finally, some factors that can make heat pump drying cost effective and energy efficient were elucidated. Adoption of heat pump drying technology for drying of fruits and vegetables in sub-Saharan Africa will improve product quality and reduce energy consumed in the process.

## Figures and Tables

**Table 1:** Comparison of HPD with other commonly used dryers.

Item	HPD drying	Hot air drying	Vacuum drying	Freeze drying
SMER (kg/kWh)	1.0–4.0	0.1–1.3	0.7–1.2	0.4 and lower
Operating temperature range (°C)	−10 to 80	40 to very high	30–60	−35 to >50
Operating % RH range	10–80	Varies depending on temperature	Low	Low
Drying efficiency (%)	Up to 95	35–40	Up to 70	Very low
Drying rate	Faster	Average	Very slow	Very slow
Capital cost	Moderate	Low	High	Very high
Running cost	Low	High	Very high	Very high
Control	Very good	Moderate	Good	Good

Source: Mujumdar and Jangam [20].
